# Co-teaching in an Undergraduate Clinical Skills Course: Physicians and Social Behavioural Scientists Use a Shared Mental Model to Highlight Complementary Aspects of Medical Interviewing and Physical Exam Skills

**DOI:** 10.15694/mep.2019.000211.1

**Published:** 2019-11-22

**Authors:** Emily Hogikyan, Jennifer Stojan, Karri Grob, Willem de Grave, Patricia Mullan, Michelle Daniel

**Affiliations:** 1 Ann and Robert H. Lurie Children’s Hospital; 2 University of Michigan Medical School; 3 Maastricht University

**Keywords:** co-teaching, clinical skills, shared mental models

## Abstract

This article was migrated. The article was marked as recommended.

**Introduction:** Interdisciplinary co-teaching by physicians (MD) and social behavioural scientists (SBS) has emerged as an innovative teaching practice in clinical skills courses, but little is known about how co-teachers operationalize instruction. The purpose of this study was to explore the shared mental model of co-teachers concerning medical interviewing and physical examination instruction.

**Methods:** Twelve individual semi-structured interviews were conducted at Brown University. Participants were asked, “ *What* and *how* do MD and SBS faculty contribute to teaching medical interviewing and physical examination skills?” Transcripts were subjected to thematic analysis. Discourse analysis was also used to determine if what faculty individually described as contributing to instruction was observed by the co-teacher.

**Results:** Physician and SBS faculty emphasized different but complementary aspects of medical interviewing and physical examination skills. Physicians focused on *content,* targeting clinical reasoning, differential diagnosis, economy of movement, efficiency, synthesis, and technical skills. SBS faculty focused on *process,*emphasizing active listening, presence, non-verbal communication, rapport building, empathy, and patient comfort.

**Discussion:** Co-teachers consistently articulated their relative contributions to teaching medical interviewing and physical examinations. Their shared mental model emphasized the importance of both *content* and *process*, creating a learning environment supporting the development of both biomedical and patient-centred perspectives.

## Introduction

Graduating medical students must demonstrate proficiency in clinical skills as well as medical knowledge. In 2002, the Association of American Medical Colleges (AAMC) published a survey showing that only 24% of United States and 52% of Canadian medical schools had formal clinical skills curricula ( Corbett and Whitcomb, 2004). These statistics have since rapidly changed, driven in part by national assessments such as the United States Medical Licensing Examination (USMLE) Step 2 Clinical Skills Exam and National Board of Osteopathic Medical Examiners (COMLEX) Level 2 Performance Evaluation ( Gilliland *et al.,* 2008). Today, nearly all US, Canadian, and international medical schools have formal clinical skills courses.

Clinical skills courses typically include instruction on medical interviewing, physical examination skills, oral presentations, written documentation, clinical reasoning, and sociobehavioural topics. In 2008, the AAMC published competencies for pre-clerkship clinical skills education that included fostering the ability to engage and communicate with patients, take a clinical history, and perform a physical examination ( Corbett *et al.,* 2008). More recently, the AAMC published the Core Entrustable Professional Activities (EPAs) for Entering Residency ( Englander *et al.*, 2016). Notably, the first EPA describes the ability to perform a history and physical exam as both a “data gathering and patient interaction activity,” that encompasses *content* (e.g. relevance, organization, completeness, and accuracy) and *process* (e.g. patient-centredness) aspects of these skills ( Englander *et al.*, 2016).

Physicians are often assumed to have the necessary *content* and *process* knowledge to teach medical students medical interviewing and physical examination (PE) skills. In many clinical skills courses, physicians are the sole educators of medical students. However, the literature is filled with reports of physicians failing to use patient-centred history and PE techniques ( Joshi, 2015; Stewart, 1995). Thus, one might question, are physicians alone the best teachers of these skills? In one survey, first year medical students perceived both physicians and behavioural scientists as effective clinical skills teachers, however behavioural scientists were thought to be better teachers overall due to the formal training they have in subjects such as human behaviour ( Quirk and Letendre, 1986). While these non-physicians may be more equipped to teach patient-centred *processes,* they may not be as well-equipped as physicians to teach the biomedical *content.*

Some medical schools have adapted innovative instructional models to address this problem. One model used at several institutions involves having both physicians and social behavioural scientists (SBS) co-teach ( Wilkes *et al.*, 1998; Taylor *et al.*, 2012). Co-teaching involves two professionals with complementary expertise providing instruction, with equal responsibility for the planning, conduction, and assessment of a group of students ( Cook and Friend, 1995). At the Warren Alpert Medical School of Brown University, social behavioural scientists (SBS) co-teach with physician faculty, providing longitudinal small group instruction to first and second-year medical students in their clinical skills course known as Doctoring. Through their interactions with each other and with students, the co-teachers create an environment that models their values and diverse perspectives. Learning is socially constructed ( Kay and Kibble, 2016) in this interdisciplinary context and may result in skill development that is more balanced between *content* and *process.*

Co-teaching in clinical skills courses has been largely unexplored. Further investigations into the process and outcomes studies are needed. At present, little is known about how co-teachers operationalize instruction of the medical interview and physical examination. In *Teacher Perspectives of Interdisciplinary Co-Teaching Relationships in a Clinical Skills Course: A Relational Coordination Theory Analysis*, the parent study to the current analysis, the authors described what teachers perceive as influencing the quality of co-teaching relationships ( Daniel *et al.* 2018). The authors identified having *shared knowledge and understanding* as one critical component of effective co-teaching relationships ( Daniel *et al.* 2018). To function as an effective team, MD and SBS ideally should share a common understanding of their respective roles and their contributions to the development of medical students’ clinical skills. In other words, they should have a shared mental model ( Canon-Bowers, Salas and Converse, 2009). Shared mental models have been shown in multiple contexts to support effective teamwork ( Westli *et al.*, 2010), and to influence learning and performance ( Floren *et al.*, 2018).

The purpose of the present study is to explore in detail the shared mental model of interdisciplinary co-teachers surrounding medical interviewing and physical exam instruction of pre-clinical medical students. Teacher perceptions will be used to help us better understand *what* and *how* MD and SBS faculty contribute to the instruction of these core skills, to help guide decisions about faculty composition and pedagogy in clinical skills courses.

## Methods

We embraced a constructivist world view, using both thematic content analysis ( Braun and Clarke, 2014) and discourse analysis ( Kärreman and Levay, 2017) to understand “truths” constructed by and between co-teachers and the researchers. We chose qualitative methods to examine the shared mental model of interdisciplinary co-teachers, as prior studies have shown these to be effective techniques to aid understanding of complex phenomena ( Floren *et al.*, 2018). The use of discourse analysis in particular allows us to evaluate important properties of the shared mental model, namely *similarity*(e.g. how much overlap exists between team members’ mental models) and *accuracy* (e.g. how consistent is the shared mental model with an “ideal” model) ( Floren *et al.*, 2018; Mathieu *et al.*, 2000).

*Context:* Co-teaching has been in use at Brown University for nearly two decades, thus providing an ideal environment to study the process. In Doctoring, co-teachers are responsible for the longitudinal instruction and assessment of a small group of eight pre-clinical medical students (3 hours per week for 18 months). Co-teachers utilize locally developed medical interviewing and physical exam checklists that integrate content and process to guide their instruction ( **Supplementary File 1**). Teaching occurs in both the classroom and in simulated clinical environments using standardized patients. Co-teachers use both team-teaching, where they deliver instruction together, as well as station teaching, where students rotate between instructors and learning activities ( Cook and Friend, 1995). Objective Structured Clinical Examinations (OSCEs) are the primary means of summative assessment, and OSCEs are jointly evaluated by physician and SBS faculty.

*Subjects and Sampling:* A total of sixty-fourfaculty co-teach in Doctoring, 32 SBS and 32 physicians. Faculty sampling for this study was purposive to ensure that characteristics including age, gender, specialty, and number of years co-teaching were representative of the faculty composition of the course. Participants were recruited by email or in person, informed of the purpose of the study and provided written consent. All faculty asked to participate agreed. Participation was completely voluntary.

*Data Collection and Data Analysis:* A single researcher, who was a Doctoring course director and former co-teacher (M. Daniel), conducted twelve individual semi-structured interviews, six with SBS and six with physician faculty, using open-ended questions ( Appendix A). Interviews were audio-recorded and transcribed verbatim.Transcripts were analysed for themes using a six-step framework for thematic analysis ( Clarke and Braun, 2013). In the parent study, grounded theory was used to construct a theory or framework of collaborative teaching ( Daniel *et al.* 2018). The present study did not aim to build theory, so these alternative analytic procedures were selected. M. Daniel and E. Hogikyan familiarized themselves with the data and generated initial codes. They then searched for themes that aided understanding of the shared mental model. Review and refinement of themes continued until final agreement was reached. The final coding scheme was entered into NVivo ^TM^ for data management. Discourse analysis was utilized to determine if what SBS and physician faculty individually described as their contribution to instruction is what was observed by the other. Themes were synthesized into a conceptual model for co-teaching clinical skills. The final themes and interpretations were presented to two SBS and two MD participants for member-checking in order to verify the accuracy and representativeness of the account.

## Results/Analysis

Four male and two female physician faculty, ages 36 to 78, participated in the study. MD faculty had been co-teaching in the course for 2-9 years. Specialties represented included internal medicine, emergency medicine and family medicine. Five female and one male SBS faculty, ages 39 to 78, participated. SBS faculty had been co-teaching for 1-8 years. Disciplines represented included psychology, social work, counselling and nursing. Co-teachers in the sample typically worked with the same partner 1-2 years before changing partners. One physician and one SBS in the sample worked with the same partner for 8 years. In the physician sample, gender was slightly skewed towards males and in the SBS sample, age was slightly skewed towards older faculty. The sample was otherwise representative of the faculty composition of the course. Quotations (Q) were identified with anonymous codes for physicians (MD1-6) and social behavioural scientists (SBS1-6).

Physicians and SBS emphasized different, but complementary aspects of the medical interview and physical examination in their instruction and feedback on these skills. Overall, SBS faculty tended to focus on patient-centred *processes* (the “how”) and physician faculty focused on the biomedical *content* (the “what”) aspects of the medical interview and physical exam. Table 1 illustrates the differences in perspective and feedback provided by co-teachers, showing themes distilled from representative quotes.

**Table 1.** Social Behavioural Scientist and Physician Themes with Representative Quotes

SBS Themes Process or “How?”	Representative Interview Quotes	Physician Themes Content or “What?”
**Active** **Listening and Presence**	**Medical Interview** **Q3:** “For (MD) it’s bringing together that differential diagnosis, forming the synthesis of what you’re learning from the patient as you’re performing the interview, the wheels are turning in your head, trying to calculate what is going on, what diagnostically, what tests, what possibilities, what other information is needed, ...I guess for me it’s that mindfulness of listening, active listening to the patient. To listen, for the student to just be present in that moment with that person, ... of knowing there is an actual person in front of you. You have to pay attention to that. That’s where your focus is.” (SBS1)	**Clinical Reasoning and ** **Differential Diagnosis**
**Non-Verbals** **and Empathy**	**Medical Interview** **Q4:** “When they ask the questions, how they’re going to use that information to ask the next questions...what am I thinking about for diagnosis? How can I ask more questions to help eliminate or support that? And try not leave out the important things... (the SBS looks at) more of how the student is asking the questions and body language... and reinforcing students to giving feedback to the patient that shows compassion and empathy. I think that’s what they’re looking for.” (MD5)	**Hypothesis Driven ** **Interview Content and Relevance**
**Patient Comfort**	**Physical Exam** **Q5:** “I’m looking for economy of movement, about performance of a move or technique...(SBS) will notice some little things that I will not necessarily pick up on sometimes... “I like how you held their hand when you were picking them up from the table. You patted them on the shoulder...” I think it helps them to know that it is actually seen, recognized and important, whereas I am looking- did they place the stethoscope properly... or are they just going through the motions? ... The things that (SBS) points out are things that matter to people.” (MD1)	**Economy of Movement and Efficiency**
**Connection, Rapport Building and Empathy**	**Physical Exam** **Q6:** “I think the physician ends up...instructing a little bit on the physical exam, the efficiency...These are things that, it just takes practice and experience to know the phrases that are going to be the most appropriate, useful and efficient for asking a patient to perform a certain manoeuvre, so those are communication elements I think the physician still does better... (SBS) might notice things about draping. She will notice if it just feels a little bit awkward to her... the mechanics of it.... the connection with the patient, like, ‘Did it feel warm or didn’t it feel warm’, really... Honestly I think it’s an element of warmth, I think on the part of the SBS, like that’s what they like to see the encounter imbued with because that’s mostly what they’re watching for.” (MD2)	**Technical Skill and Knowledge**

MD2 succinctly described the dichotomy. “I think what (the SBS) may bring a little bit more to the medical interview is ‘Ok, so this is the information you want. Well *how* (emphasis) exactly do you go about getting it?’ Whereas what I think I bring a little bit more is, ‘ *What* information do you want?’” ( **Q1**)

Within the medical interview, SBS focused on the process including active listening and presence, rapport building and empathy, and non-verbal communication ( Table 1, **Q3**, **Q4**). In contrast, the physicians focused on the content of the interview, including relevant questions and follow-up questions, organization, and clinical reasoning ( Table 1, **Q3**, **Q4**).

SBS2 described the different perspectives of the faculty for the physical exam. “MD was keying more on the detailed aspects of performing exams properly..I was looking through the eyes of the patient. How eye-contact, non-verbal cues, proximity, gesticulations, head nods, posture - how are you connecting to your patient?” ( **Q2**)

Within the physical exam, SBS again focused on the “how”, targeting awareness of the patient’s perspective, vulnerability and comfort, connecting with the patient, and non-verbal communication ( Table 1, **Q5**, **Q6**). Physicians emphasized proper technique, organization, efficiency or economy of movement, selecting relevant manoeuvres (e.g. hypothesis-driven), clinical reasoning, and interpretation of findings.

From Table 1 and the above quotes, we can see that faculty consistently articulated a *similar* shared mental model of their contributions to instruction with significant overlap in their descriptions, regardless of if MD or SBS faculty were describing the construct. They also articulated an *accurate* shared mental model, as the content and process components that both faculty described align with integrated features of the course checklists ( **Supplementary File 1**).

Two SBS and two MD faculty reviewed the themes and shared mental model constructed by the participants and researchers and confirmed that they were representative of their experience.

## Discussion

Throughout the interviews, participants’ accounts of instruction and feedback showed the unique contributions of MD and SBS faculty to teaching the medical interview and physical exam, and clearly demonstrated that faculty have a *similar* and *accurate* shared mental model of instruction. The MD faculty focused on the “what”, while the SBS faculty focused on the “how”. Together, MD and SBS co-teachers operationalized instruction using a shared mental model that emphasized *both* physician-centred, as well as patient-centred, components of clinical skills.

Sociolinguistic theory helps us validate our research process and findings. Sociolinguistic theory states that context, cultural norms, and expectations influence the way we speak and the language that we use ( Denning, 1973). In our discourse analysis, we relied on the open and honest expression of language in individual interviews to describe the phenomenon of co-teaching clinical skills. The co-teachers described their contributions to instruction in similar ways and MD and SBS faculty closely aligned in how they described the *what* versus *how* paradigm ( Table 1). This indicates that the research conditions allowed for an accurate description of the shared mental model and the study methods produced results centred on the same themes, regardless of which co-teacher was describing the phenomenon. The overlap in themes provides evidence of the *similarity* of the shared mental model, a key component of effective teamwork ( Floren *et al.*, 2018). Our discourse analysis confirmed that these two professionals provided significantly *different* instruction and feedback to medical students. These findings help us better understand the learning environment created and modelled in co-taught classrooms - one which fosters the expression of complementary expertise and provides holistic instruction and feedback on both biomedical and patient-centred components of skills.

Of note, several models have been developed that integrate the biomedical and patient perspective to guide instruction in clinical skills courses ( Silverman, 2014). For example, the Disease-Illness model interleaves two parallel frameworks, the disease framework (physician perspective) and the illness framework (patient perspective) ( Pendleton, 1984). The well-accepted enhanced Calgary-Cambridge framework integrates content (structure, organization, flow) with process (building the relationship, non-verbals, rapport, shared decision making) ( Kurtz, *et al.,* 2003). In the Doctoring curriculum, locally developed medical interviewing and physical exam checklists are utilized that integrate content and process. Regardless of which model of integration is used, faculty may have strong professional tendencies to emphasize some components of the model over others. We observed this in co-teachers’ reports of their instruction and feedback. In the interviews, physicians noted the difficulty of providing comprehensive comments and felt the need to prioritize their feedback on the biomedical content. One physician even noted that their SBS co-teacher provided feedback that they alone would likely not have given ( **Q5**). This physician went on to note that the presence of the SBS faculty ensured emphasis on the things that mattered to patients. The different contributions of MD and SBS faculty to the learning environment appeared to provide a balanced view, integrating both perspectives. A key component of shared mental models is their *accuracy*, or how well they align with an “ideal” ( Floren *et al.*, 2018). This study gives us confidence that the instruction operationalized by MD and SBS aligns well with integrated models described in the literature ( Silverman, 2014; Pendleton, 1984) and with the course checklists.

When learning is viewed as a socially constructed process ( Palincsar, 1998), the value of the MD-SBS combination becomes even more apparent: medical students’ professional development is socially situated within their Doctoring small groups. Their knowledge is constructed through interactions with their interdisciplinary faculty and peers. By modelling diverse values and perspectives, co-teachers created an environment that emphasized both the biomedical *content* and patient-centred *process* components of the medical interview and physical examination. If learners were exposed to only a single instructor (e.g. MD or SBS), or a single profession (e.g. co-teaching pairs comprised of two physicians or two social behavioural scientists), the learning environment may be skewed towards the values of that profession, and the instruction operationalized by faculty may not align as well with the “ideal”. Further research is needed to determine how accurately these other combinations of faculty operationalize instruction.

The findings of this study have practical implications. For institutions already using interdisciplinary co-teaching for clinical skills instruction, this study provides evidence that MD-SBS faculty pairs operationalize instruction in a manner that emphasizes both *content* and *process* using a *similar* and *accurate* shared mental model. For institutions implementing new clinical skills courses, revising Doctoring curricula, or considering changing the faculty composition of their courses, this study suggests interdisciplinary co-teaching is a model to strongly consider, as co-teachers may provide a more balanced and integrated learning environment than physicians or SBS faculty can create alone.

Depending on the institution, it may be challenging to recruit qualified SBS faculty. Faculty and student buy-in, for teaching with and learning from allied health professionals, may also be an obstacle to overcome. Furthermore, there may be financial implications: having physicians teach alone may be less expensive than MD-SBS co-taught classrooms, however, courses who currently use two physicians to teach may experience a cost savings. Of course, making a change to co-taught classrooms may require significant restructuring of curricula and faculty development.

### Limitations

The results of this study should be interpreted with its limitations in mind: shared mental models can be difficult to elucidate, as researchers must rely on the expression of what is inherently an internal cognitive structure. Although qualitative methods are well accepted techniques for evaluating shared mental models ( Floren *et al.*, 2018), these methods are inherently context specific, limiting the generalizability of the findings. Furthermore, this study only explored teacher perspectives, and did not account for student perspectives or learner outcomes. Future research in this area is needed. Students are active participants in co-taught classrooms, and they construct knowledge based on social interactions. We do not know how learners assimilate information from MD and SBS faculty. Learners themselves may differentially weight instruction and feedback, giving more emphasis to the biomedical perspective, which they may view as more concordant with their professional identity formation.

## Conclusion

Physician and SBS faculty appear to have a shared mental model of co-teaching instruction, as they consistently articulate their own and each other’s relative contributions to teaching medical interviewing and physical exam skills. This study’s results give us confidence that interdisciplinary co-teachers predictably deliver complementary instruction on both biomedical *content* andpatient-centred *processes.* Physician trainees must develop skills that allow them to arrive at evidenced-based differentials with technical precision while being patient-centred. This study suggests having both physician and SBS faculty promotes instruction in a holistic manner, emphasizing the importance of both.

## Take Home Messages

• Interprofessional co-teaching between physicians and social behavioural scientists may optimize instruction of the medical interview and physical exam.
• Physicians and social behavioural scientists (SBS) have a shared mental model of instruction that emphasizes different but complementary aspects of these skills.
• Physicians focus on the biomedical *content* or the “what” (e.g. interview content, clinical reasoning and differential diagnosis, technical skills and knowledge).
• SBS focus on the patient-centred *process* or the “how” (e.g. active listening, presence, non-verbal cues, empathy, connection and rapport building).
• Together, they promote holistic instruction that honours the importance of both.

## Notes On Contributors

Emily Hogikyan, is a Paediatrics Resident, Ann and Robert H. Lurie Children’s Hospital, Chicago, Illinois.

Jennifer Stojan MD, MHPE is Director of Doctoring and an Associate Professor in the Departments of Internal Medicine and Paediatrics, University of Michigan Medical School, Ann Arbor, Michigan.

Karri Grob, EdS, is the Assistant Director of Evaluation and Assessment at the University of Michigan Medical School in Ann Arbor, Michigan.

Willem de Grave, PhD is a Senior Lecturer in the Department of Educational Development & Research at Faculty of Health, Medicine and Life Sciences (FHML), Maastricht University in Maastricht, The Netherlands.

Patricia Mullan, PhD, is Professor in the Department of Learning Health Sciences at the University of Michigan Medical School in Ann Arbor, Michigan.

Michelle Daniel, MD, MHPE is Assistant Dean of Curriculum, and an Associate Professor in the Departments of Emergency Medicine and Learning Health Sciences at the University of Michigan Medical School in Ann Arbor, Michigan.

## Appendices

### Appendix A:

Interview Guide

Background Questions / Icebreaker:

• How old are you?
• What is your (medical / SBS) specialty?
• How many years have you been a co-teacher?
• How many co-teaching partners have you had?
• How long have you been with your current co-teaching partner?

Questions of interest:

• What does the physician faculty contribute to learning the medical interview?
• What does the SBS faculty contribute to learning the medical interview?
• How does the physician faculty contribute to learning the physical exam?
• How does the SBS faculty contribute to learning the physical exam?
