# Supplementary material for: Co-teaching in an Undergraduate Clinical Skills Course: Physicians and Social Behavioural Scientists Use a Shared Mental Model to Highlight Complementary Aspects of Medical Interviewing and Physical Exam Skills
Source: MedEdPublish (2016). 2019 Nov 22;8:211. [Version 1] doi: 10.15694/mep.2019.000211.1 (PMC10712534; doi:10.15694/mep.2019.000211.1)
Supplement: Peer review history

# Reviewer report: Reviewer response for version 1

**DOI:** 10.21956/mep.19937.r30251

**Published:** 2019-12-11

**Authors:** Sharon Obadia

**Affiliations:** 1 A.T. Still University

This review has been migrated. The reviewer awarded 5 stars out of 5 This study clearly and skillfully demonstrates the value of varied perspectives when approaching the teaching of clinical skills to pre-clinical learners. The notion that the physician teacher can (or should) do it all is dispelled here. This article provides needed evidence of the benefit of physician and SBS faculty co-teaching that stakeholders of clinical skills courses can bring to their administrators to improve teaching methods already in place or in creating a new clinical skills program.

Reviewer Expertise:

NA

No decision status is available

# Reviewer report: Reviewer response for version 1

**DOI:** 10.21956/mep.19937.r30252

**Published:** 2019-11-26

**Authors:** Subha Ramani

**Affiliations:** 1 Harvard Medical School, Brigham and Women's Hospital

This review has been migrated. The reviewer awarded 5 stars out of 5 This is an important article that separates out the content and process of patient communication skills and makes a cogent argument for the value of co-teaching by physicians and social behavioral scientists. The co-teaching curricula described are innovative, the study methodology is appropriate to answer study questions and the results are illuminating. The article is very well written and well referenced.Even though this curriculum pertains to clinical skills teaching, the results can have an impact on co-teaching models in any field.The only comments I would add are: the first paragraph in the introduction seems unnecessary and starting with communication skills would have sufficed.In the discussion section, several models and theories are discussed. While all are relevant to the findings and messages, readers should take time to tease out what the authors are trying to say within each paragraph- sociolinguistics, various communication models, shared mental models, social construction.All educators who design communication skills curricula will find this useful. In addition, all educators considering co-teaching as a model can learn lessons from this study.I enjoyed reading this article very much and have take home points.

Reviewer Expertise:

NA

No decision status is available

# Reviewer report: Reviewer response for version 1

**DOI:** 10.21956/mep.19937.r30253

**Published:** 2019-11-23

**Authors:** Felix Silwimba

**Affiliations:** 1 University of Lusaka

This review has been migrated. The reviewer awarded 5 stars out of 5 I enjoyed reading through the article. it addressed my concerns on doctoring training. i think it is worth exploring by medical educators in low-middle income countries.

Reviewer Expertise:

NA

No decision status is available
